# Optimal Mapping of Spiking Neural Network to Neuromorphic Hardware for Edge-AI

**DOI:** 10.3390/s22197248

**Published:** 2022-09-24

**Authors:** Chao Xiao, Jihua Chen, Lei Wang

**Affiliations:** The College of Computer Science, National University of Defence Technology, Changsha 410000, China

**Keywords:** spiking neural network (SNN), neuromorphic computing, internet of things (IoT), energy efficiency, mapping

## Abstract

Neuromorphic hardware, the new generation of non-von Neumann computing system, implements spiking neurons and synapses to spiking neural network (SNN)-based applications. The energy-efficient property makes the neuromorphic hardware suitable for power-constrained environments where sensors and edge nodes of the internet of things (IoT) work. The mapping of SNNs onto neuromorphic hardware is challenging because a non-optimized mapping may result in a high network-on-chip (NoC) latency and energy consumption. In this paper, we propose NeuMap, a simple and fast toolchain, to map SNNs onto the multicore neuromorphic hardware. NeuMap first obtains the communication patterns of an SNN by calculation that simplifies the mapping process. Then, NeuMap exploits localized connections, divides the adjacent layers into a sub-network, and partitions each sub-network into multiple clusters while meeting the hardware resource constraints. Finally, we employ a meta-heuristics algorithm to search for the best cluster-to-core mapping scheme in the reduced searching space. We conduct experiments using six realistic SNN-based applications to evaluate NeuMap and two prior works (SpiNeMap and SNEAP). The experimental results show that, compared to SpiNeMap and SNEAP, NeuMap reduces the average energy consumption by 84% and 17% and has 55% and 12% lower spike latency, respectively.

## 1. Introduction

Internet of things (IoT), an emerging computing paradigm, integrates various sensors over a wireless network. The traditional IoT transfers the collected data by sensors to the cloud. However, with an increase in the number of IoT devices, it becomes difficult to centrally process the collected data in the cloud for a variety of reasons, such as the massive workload on the IoT network, latency, and privacy concerns [1]. Edge computing moves the data processing from the cloud to the edge nodes close to the sensors. The data collected by sensors can be processed locally or transferred to the cloud after the local preprocessing. An artificial neural network (ANN) has been deployed on IoT devices to perform special tasks such as voice recognition and verification [2]. However, the intensive memory and processing requirements of conventional ANNs have made it difficult to deploy deep networks to resource-constrained and power-constrained IoT devices.

The spiking neural network (SNN), known as the third generation of the neural network, has been introduced into many application fields including electrocardiogram heartbeat classification [3], object recognition [4], waveform analysis [5], odor data classification [6], and image classification [7]. SNN has the potential to effectively process spatial-temporal information. Compared with ANN, SNN has the characteristics of lower power consumption and smaller computation load. Neurons in an SNN communicate with each other by sending spikes across synapses. A spiking neuron accepts spikes from its presynaptic neurons and integrates the corresponding weights to update its membrane potential. A neuron fires a spike when its membrane potential reaches the firing threshold and then the membrane potential is reset.

Neuromorphic hardware, the new generation of brain-inspired non-von Neumann computing system, has the potential to perform complex computations with less memory footprint, more energy efficiency, and faster than conventional architectures. Neuromorphic hardware implements spiking neurons and synapses, which makes them suitable for executing SNN-based applications. Recently, many neuromorphic processors have been developed, such as TrueNorth [8], Loihi [9], SpiNNaker [10], Unicorn [11], and DYNAPs [12]. Zhang et al. [13] propose a scalable, cost-efficient, and high-speed VLSI architecture to accelerate deep spiking convolution neural networks (SCNN). The neuromorphic hardware typically consists of multiple cores and each core can only accommodate a limited number of neurons. For example, the TrueNorth includes 4096 neurosynaptic cores and a single core has 256 axons, a 256 × 256 synapse crossbar, and 256 neurons. To support the inter-core communication, the Network-on-Chip (NoC) [14] is adopted as an interconnecting mechanism in the neuromorphic hardware.

Before an SNN is executed on neuromorphic hardware, the neurons of the SNN should be assigned to the target neuromorphic hardware. This step is typically segmented into two substeps. First, a large-scale SNN is partitioned into multiple clusters so that the number of neurons per cluster does not exceed the capacity of a single neuromorphic core. Second, it selects appropriate cores for the execution of clusters present in the partitioned SNN-based application.

Recently, numerous methods [15,16,17,18,19,20] have been proposed to map SNN-based applications to neuromorphic hardware. PACMAN [15] is proposed to map SNNs onto SpiNNaker. Corelet [16] is a proprietary tool to map SNNs to TrueNorth. Some general-purpose mapping approaches [17,18,19,20] employ heuristic algorithms [21,22,23] to partition an SNN into multiple clusters, with the objective of minimizing the spike communication between partitioned clusters. After the partition, they use meta-heuristic algorithms [21,24] to search for the best cluster-to-core mapping scheme.

**Figure 1:**
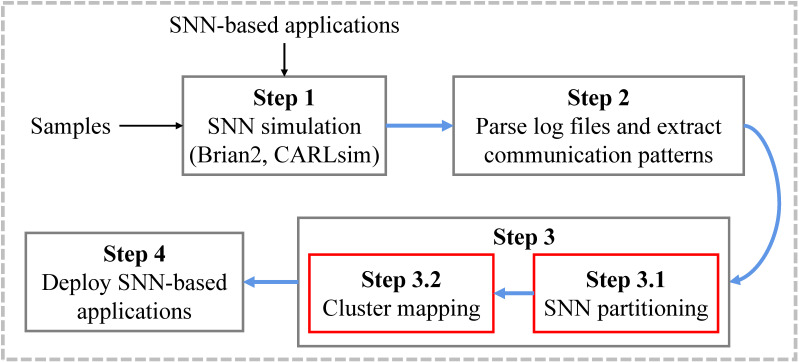
High-level overview of SpiNeMap [19] and SNEAP [20].

Figure 1 shows the high-level overview of some existing SNN mapping approaches. Before the partitioning stage, those methods need to simulate an SNN, using SNN software simulators such as Brian2 [25] and CARLsim [26], to statistically obtain communication patterns (i.e., the spike times of all neurons). Before the simulation, researchers should build the given SNN using the application programming interfaces (APIs) of the specific simulator, which may be challenging for researchers who are unfamiliar with the simulator. In addition, it will spend lots of time simulating a large-scale SNN on a software simulator. The simulation process is also included in PSOPART and NEUTAMS.

The second limitation of prior works is that they treat an SNN as a graph and partition the entire graph into multiple clusters directly, ignoring the characteristic of synapses. Exploiting the characteristics of synapses can further reduce the spike communication between clusters. The third limitation is that they always search for the best cluster-to-core scheme in the entire neuromorphic hardware, which makes them prone to trapping in the local optimum.

In this paper, we propose an efficient toolchain for mapping SNN-based applications to the neuromorphic hardware, called **NeuMap** (Optimal **Map**ping of Spiking Neural Network to **Neu**romorphic Hardware for Edge-AI). NeuMap focuses on SNN-based applications with a feed-forward topology. NeuMap first obtains the communication patterns of an SNN by calculation, instead of simulation. Based on the calculated spike firing rates, NeuMap then partitions the SNN into multiple clusters using a greedy algorithm, minimizing the communication traffic between clusters. Finally, NeuMap narrows the searching space and employs a meta-heuristic algorithm to seek the best cluster-to-core scheme. The main contributions of this paper can be summarized as follows:

(1) We study the impact of different parameters of an SNN on the spike firing rate and obtain the communication patterns of an SNN by calculation, instead of simulation. The calculation can simplify the end-to-end mapping process and get rid of challenges derived from the simulation in specific simulators.
(2) We exploit the characteristic of synapse and propose the local  partitioning strategy, which first divides the entire network into several sub-networks and partitions each sub-network into multiple clusters. The local  partitioning strategy further reduces the spike communication between neuromorphic cores.
(3) Instead of searching for the best cluster-to-core scheme across all neuromorphic cores, we reduce the searching space in advance and employ a meta-heuristic algorithm with two optimization objectives to seek the best mapping scheme. The reduction in searching space helps to avoid trapping in the local optimum.

We evaluate NeuMap with six SNN-based applications. The experimental results show that, compared to SpiNeMap and SNEAP, NeuMap reduces the average energy consumption by 84% and 17% and has 55% and 12% lower spike latency, respectively.

The remainder of this paper is organized as follows: Section 2 introduces the background and related works. Section 3 details the proposed toolchain. The experimental setup and experimental results are discussed in Section 4 and Section 5, respectively. Finally, Section 6 concludes the paper.

## 2. Background and Related Works

This section describes the theory of SNNs, the conversion of images to spike trains, and the architecture of neuromorphic hardware. The prior mapping methods are presented in this section.

### 2.1. Spiking Neural Network

**Figure 2:**
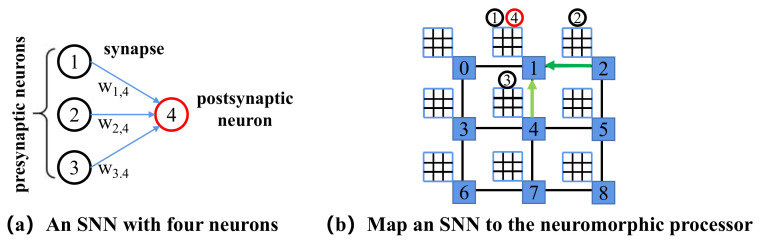
Map of an SNN to the neuromorphic hardware. (**a**) An SNN with three presynaptic neurons connected to a postsynaptic neuron. (**b**) A neuromorphic hardware has nine cores interconnected by the NoC.

SNN, as the third generation of the neural network, has attracted extensive attention because they are capable of processing spatio-temporal information with high energy efficiency in an event-driven way. The basic units of SNNs are spiking neurons and synapses interconnecting the neurons. Figure 2 illustrates an SNN with three presynaptic neurons connected to a postsynaptic neuron via synapses with weights $w_{1,4}$, $w_{2,4}$, and $w_{3,4}$, respectively. The leaky integrate-and-fire (LIF) model [27] is a kind of popular spiking neuron model to be implemented in neuromorphic hardware. The dynamics of the LIF neuron are defined as

(1) $$\tau \frac{dV(t)}{dt}=-\left(V\left(t\right)-V_{rest}\right)+X\left(t\right)$$

where V(t), X(t), $V_{rest}$, and τ are the membrane potential at time *t*, the input, the resting potential, and the membrane time constant, respectively. When the membrane potential V(t) exceeds the threshold potential, the neuron will fire a spike and then the membrane potential V(t) goes back to the resting potential.

Deep multi-layer neural networks have achieved outstanding performance in solving complex problems. To overcome the vanishing-gradient problem, Inas et al. [28] proposed OSLD, a new anti-vanishing back-propagated learning algorithm. Prior works [29] directly train SNNs using backpropagation; however, it is insufficient when training spiking architectures of the size of VGG-16 [7]. A more straightforward approach is to convert the pre-trained ANNs into equivalent-accurate SNNs [7], which has achieved a great improvement in accuracy.

### 2.2. Input Coding

SNNs use spike trains to encode the information. Rate coding is one of the most commonly used coding techniques for spikes in SNNs. In rate coding, the number of spikes fired over a period of time is counted, and the spike firing rate is proportional to the signal intensity. The spike firing rate ***r*** of a neuron can be formulated as Equations (2) and (3).

(2) $$r=\frac{\sum_{t=1}^{T}S\left(t\right)}{T}$$

(3) $$S\left(t\right)=\left\{\begin{array}{cc} 1, & if\;the\;neuron\;fires\;a\;spike\;at\;time\;t\\ 0, & otherwise. \end{array}\right.$$

**Figure 3:**
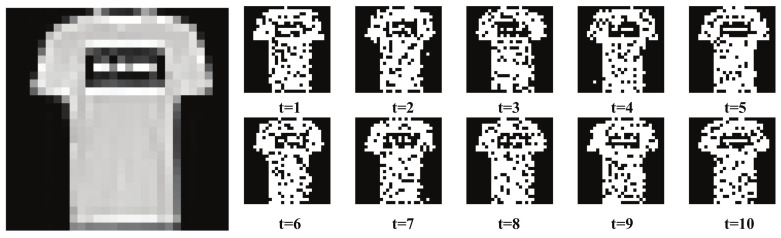
An image of Fashion-MNIST [30] dataset is presented in the form of Poisson-distributed spike trains.

Figure 3 shows an image from the Fashion-MNIST dataset [30] converted into Poisson-distributed spike trains for ten time steps. The Fashion-MNIST dataset is a collection of 28 × 28 pixel images in ten classes. Each pixel corresponds to an input neuron and the firing rate of an input neuron is proportional to the intensity of the pixel.

### 2.3. Architecture of Neuromorphic Hardware

The neuromorphic hardware is a large-scale parallel system composed of multiple computing units called neuromorphic cores. Each core implements a limited number of spiking neurons, with each neuron having a finite number of input axons. On-chip memory is used to store synaptic weights, routing tables, intermediate states, and other parameters. The computations within cores take place in parallel.

Network on chip (NoC) [14] has been extensively used as a communication framework in neuromorphic hardware due to its flexibility, scalability, and parallelism compared to traditional bus-based communication. Each core is connected to the router via the network interface (NI). The NoC transports all spikes between cores in packetized form. If a neuron fires a spike, the processor will query the routing table and obtain the addresses of destinations. Based on the query, packets with routing information, including neuron ID, source, and destination addresses, are generated and sent to NoC.

According to the topology of NoC, two types of NoC are commonly used: NoC-tree and NoC-mesh. The examples of NoC-tree are BrainScaleS [31] and DYNAPs [12]. The mesh structure with many connection channels has advantages in bandwidth and scalability. The examples of NoC-mesh include SpiNNaker [10] and TrueNorth [8].

### 2.4. Related Works

Figure 2 illustrates the mapping of an SNN with four neurons ($N_{1}$, $N_{2}$, $N_{3}$ and $N_{4}$) to the neuromorphic hardware with nine cores. $N_{1}$ and $N_{4}$ are mapped to $core_{1}$. $N_{2}$ and $N_{3}$ are mapped to $core_{2}$ and $core_{4}$, respectively. When $N_{2}$ ($N_{3}$) reaches the firing threshold and fires a spike, the processor transmits the spike to $core_{1}$ via NoC. When $N_{1}$ exceeds the firing threshold and generates a spike, there is no need to transmit the spike on NoC. If the four neurons are placed in the same core, there would be no spike message on NoC. Therefore, an optimized mapping of SNNs onto neuromorphic hardware helps to alleviate the communication pressure and reduce the performance penalty.

Prior methods generally regard an SNN as a graph, use heuristic algorithms to partition the entire SNN directly, and search for the best cluster-to-core mapping scheme across all neuromorphic cores. PSOPART [17] utilizes particle swarm optimization (PSO) [21] to partition an SNN. Both SpiNeMap [19] and NEUTRAMS [18] employ the Kernighan–Lin graph partitioning algorithm (KL) [22] to partition an SNN. The multi-level graph partitioning algorithm (ML) [23] is adopted by SNEAP [20] to partition an SNN into multiple clusters. The KL algorithm arbitrarily distributes neurons into multiple clusters while meeting the hardware resource constraints. Then, it uses three exchange strategies to swap neurons between clusters to reduce the spike messages. The ML algorithm iteratively merges two neurons with high-frequency communication into a new node, the (*coarsening step*). Then, it preliminarily partitions the SNN into *k* clusters by distributing the nodes with high-frequency communication in the same cluster, the (*initial partitioning step*). Finally, it fine-tunes the neurons to satisfy the hardware resource constraints, the (*uncoarsening step*). The ML algorithm outperforms the KL algorithm in reducing spike messages between clusters [20].

After the partition, SpiNeMap and SNEAP use the PSO and simulated annealing (SA) algorithm [24] to search for the optimal cluster-to-core mapping scheme.

## 3. Methods

**Figure 4:**
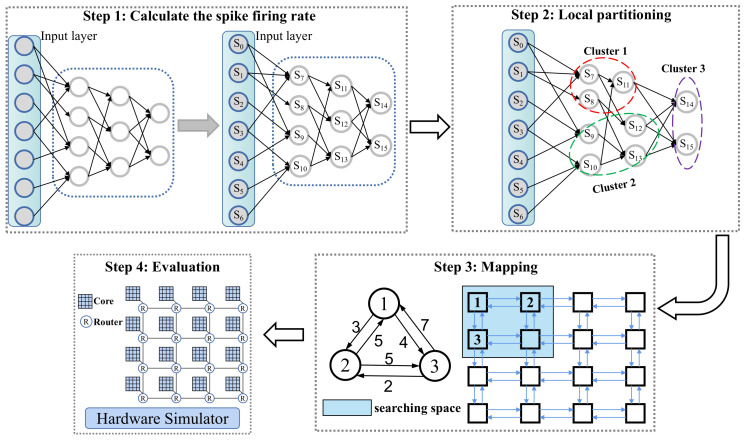
High-level overview of our proposed approach.

Figure 4 shows the high-level overview of our proposed approach which is composed of four steps, including obtaining the spike firing rates of all neurons, partitioning the SNNs, mapping the clusters to the target neuromorphic hardware, and evaluating.

For an incoming SNN-based application, NeuMap first extracts the connections and synaptic weights between neurons. NeuMap counts the spike firing times of the input neurons and then calculates the spike firing rates of other neurons. NeuMap uses a heuristic algorithm to partition an SNN into multiple clusters, minimizing the inter-cluster spike communication. By reducing the inter-cluster communication, NeuMap reduces the energy consumption and latency on NoC. Next, NeuMap uses a link congestion-aware algorithm to map the clusters to the selected cores, minimizing the hop distance that each spike message traverses before reaching its destination.

### 3.1. Calculating the Spike Firing Rates

As shown in Section 2.1, a spiking neuron accepts spikes from its presynaptic neurons, integrates the corresponding weights to update its membrane potential, fires a spike when reaching the firing threshold, and resets the membrane potential. Therefore, there are three factors affecting the firing of neurons: the synaptic weights, the external input (i.e. spikes from presynaptic neurons), and the difference between the threshold potential and resting potential. Increasing the synaptic weights or the spike firing rates of presynaptic neurons will stimulate the postsynaptic neurons to fire more frequently. On the contrary, increasing the difference between the threshold potential and resting potential reduces the spike firing times. This is because after firing a spike and resetting the membrane potential, the neuron needs to receive more spikes to reach the threshold potential again. Therefore, the spike firing rate of one neuron is positively associated with the synaptic weights and the spike firing rates of its presynaptic neurons and negatively correlated with the difference between the threshold potential and resting potential.

For an SNN-based application with *N* neurons, including *m* input neurons, we first extract the connections and synaptic weights in the network. NeuMap builds an adjacent matrix $W^{N\times N}$, where nodes are spiking neurons and edge weights between nodes are synaptic weights. In terms of the spike firing rates of all neurons, NeuMap constructs a spike firing rate vector **S**:

(4) $$S=\left\{S_{0},S_{1},S_{2}...S_{N-1}\right\}$$

S${}_{i}$ is the spike firing rate of the *i*th neuron.

**Figure 5:**
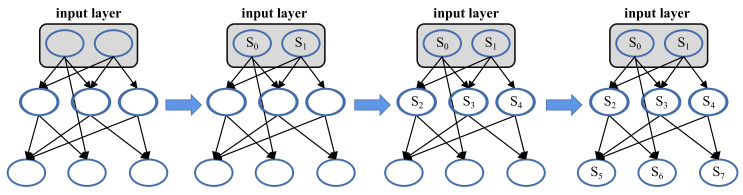
The computing process of the spike firing rates.

As shown in Figure 5, NeuMap transforms the network without recurrent connections into a tree structure. The root nodes are the neurons from the input layer. Before calculating the spike firing rate of a neuron, the spike firing rates of its all presynaptic neurons should be calculated in advance. Therefore, the calculation of spike firing rates is from up to down.

In the beginning, NeuMap counts the spike firing times of the input neurons. The representative samples from the training dataset or validation dataset are transformed into Poisson-distributed spike trains, with firing rates proportional to the intensity. NeuMap adds up the total firing times in all representative samples for every input neuron. The spike firing rates of all input neurons can be formulated as

(5) $$S_{i}=\frac{\sum_{j=1}^{K}\sum_{t=1}^{T}\left(S_{i}^{j}\left(t\right)\right)}{K\times T}\;\left(0\leq i<m\right)$$

where *K* is the number of input samples and *T* is the time step for a single sample.

After calculating the firing rates of input neurons, NeuMap calculates the spike firing rates of other spiking neurons from up to down. The computation of the spike firing rates of other spiking neurons is based on the above analysis. The computation can be formulated as

(6) $$S_{i}=\frac{\sum w_{j,i}\times S_{j}}{V_{th}-V_{rest}}\;\left(m\leq i<N,0\leq j<N\right)$$

The *j*th neuron is one of the presynaptic neurons of the *i*th neuron and w${}_{j,i}$ is the synaptic weight between the *j*th and the *i*th neuron. It should be noted that the spike firing rate cannot be more than 1 and less than 0. Therefore, when the computed firing rate exceeds 1, it will be set to 1. When the computed firing rate is less than 0, it will be set to 0. We compare the calculated spike firing rates and actual spike count in Section 5.1.

After calculating the spike firing rates of all neurons, NeuMap replaces the synaptic weights with the computed spike firing rates. For the synapse (i,j) from the *i*th neuron to the *j*th neuron, the weight is replaced by $S_{i}$. After the transformation, the given SNN is represented as a graph $G(N_{e},S_{y})$ where $N_{e}$ is the set of nodes and $S_{y}$ is the set of synapses.

### 3.2. Local Partitioning

Let $\Phi (V_{c},E)$ be the partitioned SNN with a set $V_{c}$ of clusters and a set *E* of edges between clusters. The SNN partitioning problem is transformed into G(N${}_{e}$, S${}_{y}$) →$\Phi (V_{c},E)$, which is a classical graph partitioning problem. The graph partitioning problem has been proven to be an NP-complete problem.

**Figure 6:**
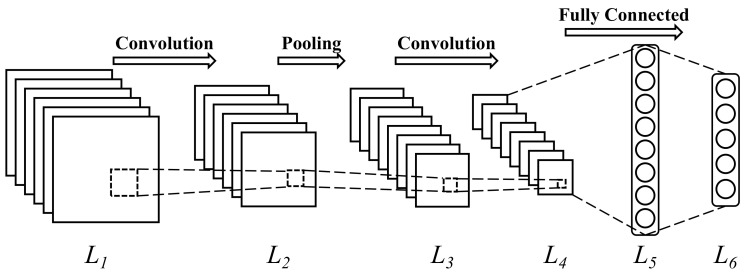
A spiking convolutional neural network with six layers.

The connections in the SNNs are localized. Take the spiking convolutional neural network, shown in Figure 6, for example. The spiking neurons in the first layer $L_{1}$ only connect to the second layer $L_{2}$. In terms of the neurons in the second layer, the presynaptic neurons are located in the first layer and the postsynaptic neurons are distributed in the third layer $L_{3}$. Therefore, the synapses are distributed in the neighboring layers.

Prior works directly partition an entire SNN, ignoring the localized connections. They traverse all neurons contained in the SNN and put neurons with high-frequency communication in the same cluster. The global searching strategy ignores the local property and often puts the neurons from multiple layers in the same cluster, which may scatter the neurons from the adjacent layers in multiple clusters.

**Figure 7:**
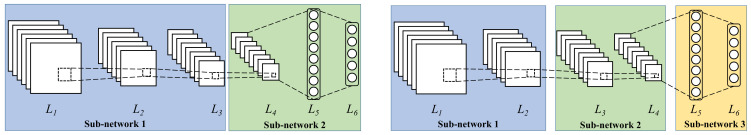
Two schemes to partition the SNN into multiple sub-networks.

Based on this knowledge, instead of directly partitioning the entire network into multiple clusters, we first divide the network into several sub-networks, shown in Figure 7. A sub-network is comprised of multiple adjacent layers. For an SNN with *L* layers, there is at least one sub-network after the division (i.e., the entire network) and at most *L* layers (i.e., every layer is treated as a sub-network). Therefore, the number of sub-networks ranges from 1 to *L*, i.e.,

(7) $$1\leq N_{sub}\leq L$$

where $N_{sub}$ is the number of divided sub-networks.

We formalize the entire partitioning process in Algorithm 1. The algorithm increases the size of sub-networks from 1 to *L* in turn and divides the entire SNN into ⌈L/size⌉ sub-networks (line 2). The variable size in the algorithm is the number of layers contained in each sub-network. After the division, only the connections between the neurons belonging to a sub-network are preserved in the sub-network. Then, we employ the multi-level graph partitioning algorithm to partition each sub-network into multiple clusters while satisfying the hardware resource constraints (lines 3–5). After partitioning all sub-networks into multiple clusters, the algorithm calculates and records the sum of spike firing rates between clusters (line 7). Finally, the algorithm selects the partitioning scheme which has the minimum spike communication between clusters (lines 9–10).
**Algorithm 1** Partitioning algorithm1:**for** size = 1 **to** L **do**2:    Divide $G(N_{e},S_{y})$ into **⌈L/size⌉** sub-networks3:    **for** each sub-network in the divided sub-networks **do**4:        Multi−level_Partition (sub-network)5:    **end for**6:    Record the partitioning result $\Phi^{size}\left(V_{c},E\right)$7:    Count and Record the sum of spike firing rates between clusters ${S_{p}^{size}}^{{}^{\prime }}$8:**end for**9:Select the minimum ${S_{p}^{i}}^{{}^{\prime }}$10:**return**$\Phi^{i}\left(V_{c},E\right)$

After selecting the partitioning scheme, we calculate the spike communication frequency between all pairs of clusters and assign it to the corresponding edge in $\Phi (V_{c},E)$.

### 3.3. Mapping

After an SNN is divided into multiple clusters, the next step is to map all the clusters to the multicore neuromorphic hardware. The NoC-based neuromorphic hardware can be represented as a graph Ψ(C,I), where *C* is the set of neuromorphic cores and *I* is the set of physical links between those cores. Mapping of an SNN onto the neuromorphic hardware is defined as a one-to-one from the set of partitioned clusters to the set of cores:

(8) $$Mapping:\Phi \left(V_{c},E\right)\to \Psi \left(C,I\right),s.t.Mapping\left(V_{i}\right)=C_{j};\forall V_{i}\in V_{c},\exists C_{j}\in C$$

**Figure 8:**
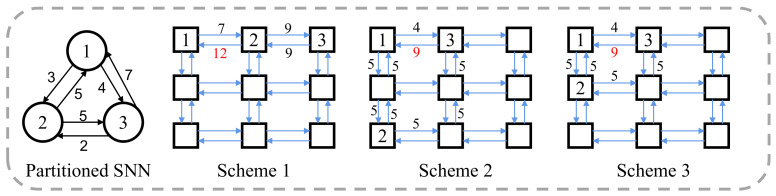
The link workload differences caused by different mapping schemes.

Different mapping schemes lead to different utilizations of interconnect segments, which impacts both energy consumption and spike latency. Figure 8 shows that an SNN has been partitioned into three clusters and the neuromorphic hardware has nine cores arranged in 3×3 mesh topology. This case uses the X–Y routing algorithm, a deterministic dimensional routing algorithm. The number attached to each edge is the sum of spike messages passing through the physical link. There are three candidate mapping schemes for the partitioned SNN on the right-hand side of Figure 8. Compared with $scheme_{2}$ and $scheme_{3}$, the maximum link workload of $scheme_{1}$ is higher. The $scheme_{2}$ and $scheme_{3}$ have the same maximum link workload. Unfortunately, compared with $scheme_{3}$, the clusters in $scheme_{2}$ are mapped to distant cores, which leads to higher spike latency and energy consumption on NoC. Therefore, the $scheme_{3}$ is the best mapping scheme among the three candidates.

Communication latency and energy consumption are two main concerns of the on-chip domain. Therefore, the spike latency and energy consumption are the most direct and effective optimization goals. Unfortunately, the evaluation of spike latency and energy consumption is time-consuming because those metrics should be obtained by simulation in the software simulator or the real hardware.

Placing communicating clusters in close proximity will decrease the energy consumption and the congestion probability on NoC. Furthermore, compared with spike latency and energy consumption, the calculation of the average hop of all spike messages takes less time. Therefore, in this paper, the average hop is adopted as one of the optimization objectives.

On the other hand, unbalanced link load distribution may cause severe local congestion on NoC such as the link from $core_{2}$ to $core_{1}$ in $scheme_{1}$. Hence, balancing link load is selected as another optimization goal in this paper. Instead of balancing the link loads directly, we minimize the maximum link load. After determining the partitioning scheme, the sum of spike messages on NoC is constant. The maximum possible number of spike messages on a single link is the sum of spike messages on NoC and the minimum is 0. Minimizing the maximum link load can narrow the gap between the maximum and minimum link loads, which balances the link load indirectly. The optimization objective of average hop can be formulated as:

(9) $$H_{min}=min\left\{H(Mapping_{i})\;i\in 1,2,...,R\right\}$$

where $H(Mapping_{i})$ is the average hop in the *i*th mapping scheme. The optimization objective of maximum link load can be formulated as:

(10) $$W_{min}=min\left\{W_{max}\left(Mapping_{i}\right)\;i\in 1,2,...,R\right\}$$

where $W_{max}\left(M_{i}\right)$ is the maximum link load in the the *i*th mapping scheme.

In this paper, we employ a meta-heuristic algorithm, the Tabu Search (TS) algorithm [32], to search for the best mapping scheme. For the partitioned SNN $\Phi (V_{c},E)$ and the target neuromorphic hardware Ψ(C,I), there are $A_{\left|C\right|}^{\left|V\right|}$cluster-to-core mapping schemes. Iterating over all mapping schemes is time-consuming, especially when the sizes of the SNN and neuromorphic hardware are large. Furthermore, when there are more alternative mapping schemes, the search algorithm is more likely to fall into the local optimum.

**Figure 9:**
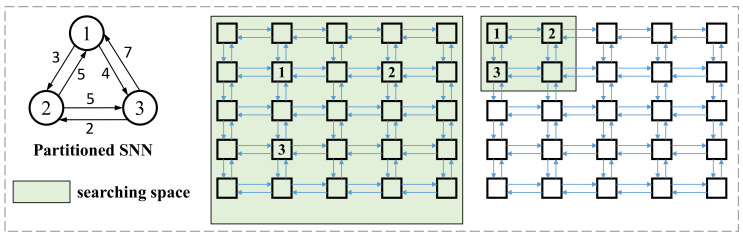
Different searching space in the mapping stage.

To avoid trapping in the local optimum, we first reduce the searching space. Figure 9 shows two searching spaces in the mapping stage. If the searching space is set as the entire neuromorphic hardware (shown in the middle subfigure), the mapping algorithm is easily trapped in the local optimum. As shown in the right subfigure of Figure 9, the searching space is set as the 2×2 square region in the upper-left corner, which contributes to a better mapping scheme. The reduction in the searching space not only helps to seek a better mapping scheme but decreases the time consumed in the mapping stage. In this paper, the searching space is set as $\left\lceil \sqrt{\left|V\right|}\;\right\rceil \times \left\lceil \sqrt{\left|V\right|}\;\right\rceil$. After setting the searching space, we use the TS algorithm, with two fitness functions $H_{min}$ and $W_{min}$, to search for the best cluster-to-core scheme.

## 4. Evaluation Methodology

In this section, we present the experiment platform. This section presents six SNN-based applications to evaluate our proposed method.

### 4.1. Experiment Platform

Unicorn [11], supporting unconstrained fan-out and flexible fan-in of neurons, is employed as the target neuromorphic hardware. Unicorn supports two spiking neuron models, the leaky-integrate-fire (LIF) and integrate-fire (IF). Unicorn contains 36 neuromorphic cores arranged in 3 × 3 C-Mesh.

To test the scalability of NeuMap, our experiment uses the hardware configuration of 8 × 8 2D-mesh NoC with the X-Y routing algorithm. The capacity of the neuromorphic core is set to 256. We first simulate the evaluated applications in different SNN software simulators using representative samples and record the spike firing information. Then, we partition the SNNs and map the partitioned clusters to the experiment platform. Finally, the spike firing information, partitioned and mapped results are used to create the routing files. Noxim++ [19], an extension version based on Noxim [33], is a cycle-accurate NoC simulator. We use Noxim++ to obtain the key performance statistics of NoC, such as energy consumption and spike latency.

We use the Python programming language to implement NeuMap. All experiments are performed on a system with the i7-10700 CPU, 16GB RAM, and NVIDIA RTX2060 GPU, running Ubuntu 16.04.

### 4.2. Evaluated SNN-Based Applications

We evaluate six SNN-based machine-learning applications: (1) image classification with the multi-layer perceptron (MLP-Fashion-MNIST) [30]; (2) handwritten digit recognition with the multi-layer perceptron (MLP-MNIST) [34]; (3) handwritten digit classification with with the LeNet-5 (LeNet-MNIST) [35]; (4) image classification with an CNN (CNN-Fashion-MNIST) with images from the Fashion-MNIST dataset [30]; (5) image classification with LeNet-5 CNN (LeNet-CIFAR10) [35] with images from the CIFAR dataset [36]; and (6) image classification with a standard CNN (CNN-CIFAR10) [7].

MLP-Fashion-MNIST and MLP-MNIST are simulated in Brian2 [25], an SNN software simulator, with the LIF model. LeNet-5-MNIST, CNN-Fashion-MNIST, LeNet-5-CIFAR10, and CNN-CIFAR10 are first trained using backpropagation. Then, we convert the four ANN-based applications into spiking neural networks using the ANN-to-SNN conversion tool SNN-TB [7]. Finally, the four converted applications are simulated in the INIsim, a built-in simulator of SNN-TB, supporting the IF model.

The topology, the number of neurons, and the number of synapses are summarized in Table 1.

## 5. Results and Discussion

In this section, we show all experimental results, including the accuracy of the calculated spike firing rates, the number of spike messages on NoC, the average hop of spike messages, the spike latency on NoC, and the energy consumption on NoC.

### 5.1. Accuracy of the Calculated Spike Firing Rates

**Figure 10:**
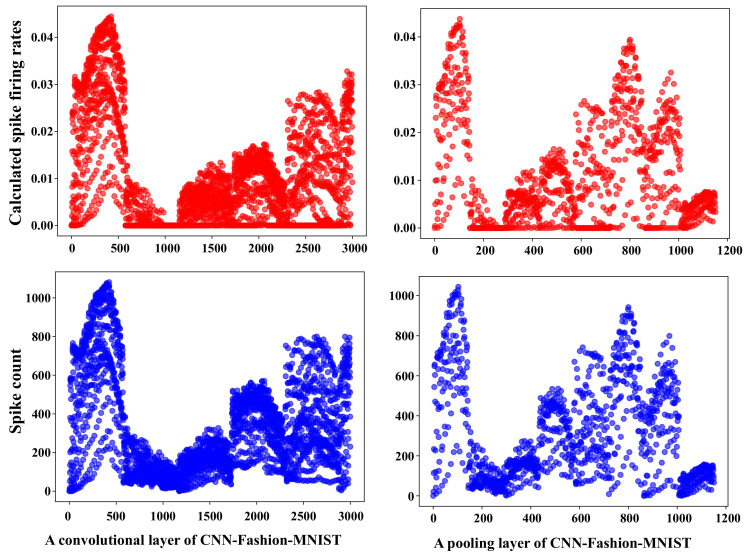
The calculated spike firing rates and actual spike count in different layers of CNN-Fashion-MNIST. The horizontal axis is the number of neurons.

**Figure 11:**
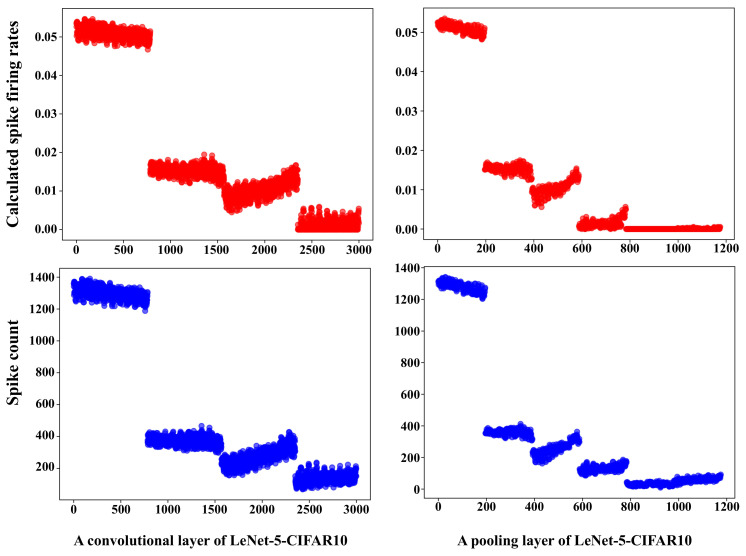
The calculated spike firing rates and actual spike count in different layers of LeNet-5-CIFAR10. The horizontal axis is the number of neurons.

We count the spike firing times at different layers from different applications when executing the applications. Figure 10 and Figure 11 show the calculated spike firing rates and the actual spike count of two different layers from two applications, CNN-Fashion-MNIST and LeNet-5-CIFAR10. It can be seen that the calculated spike firing rates are nearly consistent with the actual spike count. The precondition of accurate calculation for a neuron’s spike firing rate is that the spike firing rates of presynaptic neurons are computed accurately. Therefore, in the computing process, NeuMap first counts the actual firing times of the input neurons, which ensures the accuracy of the input layer. After that, NeuMap calculates the spike firing rates of other neurons from up to down.

Prior works, such as SNEAP, SpiNeMap, and PSOPART, obtain the communication patterns of an SNN by simulating the SNN in a software simulator. Researchers need to be familiar with the APIs of the specific simulator and reproduce the SNN before the simulation. We can get rid of the simulating process and obtain the communication patterns by calculating the spike firing rates using representative data.

### 5.2. Partitioning Performance

In the partitioning stage, all evaluated methods try to minimize the spike communication between the partitioned clusters while meeting the hardware resource constraints.

**Figure 12:**
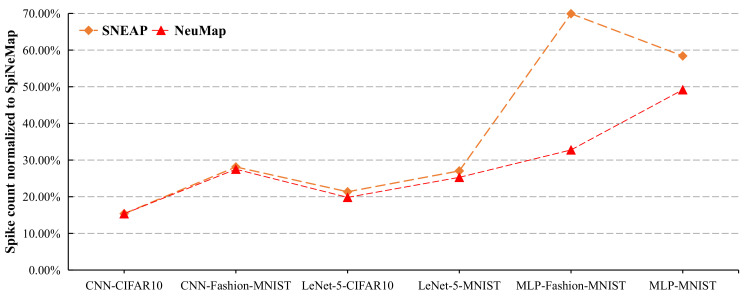
The number of spike messages on NoC normalized to SpiNeMap.

We illustrate the total number of spike messages in Figure 12. Compared with SpiNeMap, SNEAP has an average 63% lower spike count. This improvement is due to the ML algorithm outperforming the KL algorithm. The KL algorithm arbitrarily distributes neurons to *K* clusters on initialization. Next, three random exchange strategies are applied to fine tune the clusters to minimize the number of spikes between clusters. The ML algorithm iteratively folds two adjacent nodes with high-frequency communication into a new node. Compared with the KL algorithm, the ML algorithm reduces more spike messages.

Compared with SNEAP, NeuMap has an average 7% lower spike count. Both SNEAP and NeuMap partition the SNNs using the multi-level graph partitioning algorithm. Different from SNEAP, NeuMap exploits the local property of connections and divides the entire network into several sub-networks. The partition is applied to each sub-network, which avoids the dispersion of neurons from the adjacent layers. It should be noted that in both MLP-MNIST and MLP-Fashion-MNIST applications, NeuMap achieves a 9% and 37% reduction in spike messages. This is because the MLP is a kind of synapse-intensive network. The neurons of the same layer are easily distributed to many clusters when partitioning the entire network directly.

### 5.3. Mapping Performance

**Figure 13:**
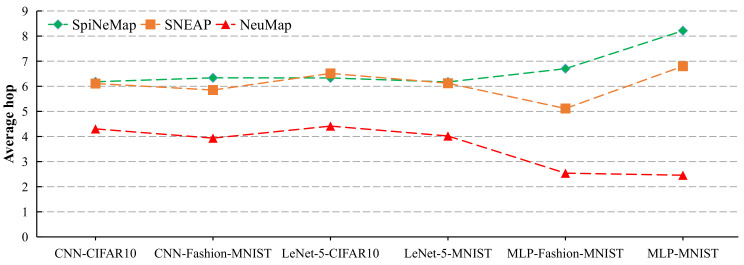
The average hop of spike messages.

The hop of one spike message is the number of routers from the source core to the destination core. We illustrate the average hop of all spike messages in Figure 13. NeuMap significantly reduces the average hop for all evaluated applications. The three evaluated methods employ different meta-heuristics algorithms to search for the cluster-to-core mapping scheme. SpiNeMap, SNEAP, and NeuMap employ the particle swarm optimization, simulated annealing, and tabu search algorithms to search for the best mapping scheme. The three searching algorithms are neighborhood search algorithms and aim to obtain the global optimum solution from the solution space. Unfortunately, as the solution space increases, the probability of those algorithms falling into the local optimum increases.

Both SpiNeMap and SNEAP treat all neuromorphic cores as the searching space, which makes them easily trapped in the local optimum. Furthermore, the probability of trapping in the local optimum is greater when the size of the neuromorphic hardware far exceeds the size of SNNs, such as MLP-MNIST and MLP-Fashion-MNIST. An effective practice to avoid trapping in the local optimum is to narrow the solution space. NeuMap sets the searching space as a $\left\lceil \sqrt{\left|V\right|}\;\right\rceil \times \left\lceil \sqrt{\left|V\right|}\;\right\rceil$ square region, which guarantees that the selected cores can accommodate all partitioned clusters. Therefore, the searching space is only related to the size of SNNs. Therefore, even if the neuromorphic cores greatly outnumber the partitioned clusters, NeuMap can search for a better mapping solution than SpiNeMap and SNEAP. The reduction in average hop improves both spike latency and energy consumption on NoC.

### 5.4. Spike Latency on NoC

**Figure 14:**
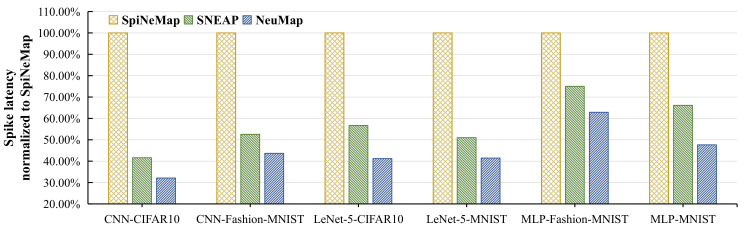
The spike latency normalized to SpiNeMap.

Figure 14 reports the spike latency of the six applications for the three evaluated approaches normalized to SpiNeMap. We make the following two observations.

First, the average spike latency of SNEAP is 42% lower than SpiNeMap. The main reason is that SNEAP reduces more spike messages than SpiNeMap, which alleviates the NoC congestion and, consequently, decreases the time to transmit the spike packets from the source core to the destination core. Second, NeuMap has the lowest average spike latency among all the evaluated methods (12% lower than SNEAP, 55% lower than SpiNeMap). There are three reasons accounting for this improvement. Firstly, NeuMap reduces the most spike messages among the three methods by using a local partitioning strategy. Secondly, in the mapping stage, NeuMap narrows the searching space, which avoids trapping in the local optimum. Thirdly, both the maximum link load and average hop are adopted as the optimization objectives in the searching process. Reducing the maximum link load can relieve the local congestion and balance the load of all physical links indirectly. Decreasing the average hop shortens the route path from the source core to the destination core. Furthermore, a short route path covers a few physical links, which decreases the congestion probability of the entire NoC.

### 5.5. Energy Consumption on NoC

**Figure 15:**
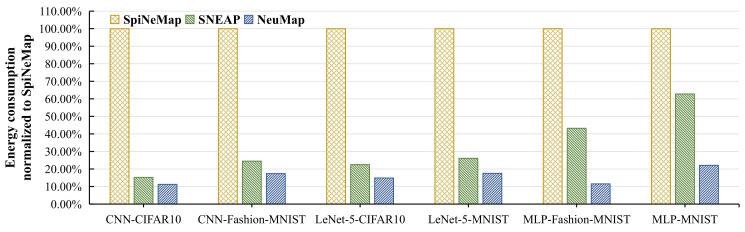
The energy consumption on NoC normalized to SpiNeMap.

This is the total energy consumption for transmitting the spike messages from the source core to the destination core. Figure 15 illustrates the energy consumption of all the evaluated applications for three proposed methods normalized to SpiNeMap. We make the following two observations.

First, SNEAP has an average 67% lower energy consumption than SpiNeMap. This reduction is because SNEAP reduces more spike messages than SpiNeMap. Second, NeuMap has the lowest energy consumption of all our evaluated approaches (on average, 84% lower than SpiNeMap, 17% lower than SNEAP). Two reasons are responsible for this improvement. Firstly, NeuMap exploits the localized connections and reduces the most spike messages among the three mapping methods. Second, NeuMap narrows the searching space before seeking the cluster-to-core mapping scheme. The reduction in searching space helps NeuMap to avoid falling into the local optimum and increases the probability of obtaining a better mapping scheme. As shown in Section 5.3, NeuMap significantly reduces the average route path of spike messages, which is the main reason for the reduction in power dissipation on NoC.

### 5.6. Performance Comparison on Recurrent SNN

Liquid state machine (LSM) was first proposed by Maass [37], and is mainly composed of the *input*, *liquid*, and *readout* layers. The synapses within the liquid layer are randomly generated and remain unchanged, leading to many recurrent connections in the liquid layer.

We use the Brian2 to create two LSM networks (800 excitatory and 200 inhibitory neurons in the liquid layer) with the LIF model. NMNIST [38] and FSDD (https://github.com/Jakobovski/free-spoken-digit-dataset, accessed on 26 July 2022), two spike-based datasets, are fed into the LSM networks, respectively.

Table 2 shows the comparison between the three mapping methods. We compare the number of spike messages on NoC under four partitioning methods, including SpiNeMap, SNEAP, NeuMap and random partition. As shown in Table 2, there are the same number of spike messages for the four partitioning methods. This is because each neuron has large numbers of connections compared to the others and there are many recurrent connections in LSM. After the partition, the post-synaptic neurons of each neuron are distributed to all clusters.

In terms of the spike latency and energy consumption on NoC, NeuMap is superior to SpiNeMap and SNEAP. This is because NeuMap narrows the solution space and searches for a better mapping scheme than SpiNeMap and SNEAP.

## 6. Conclusions and Future Work

In this work, we introduce NeuMap, a toolchain to map SNN-based applications to neuromorphic hardware. NeuMap calculates the spike firing rates of all neurons to obtain communication patterns, which simplifies the mapping process. NeuMap then makes use of the local nature of connections and aggregates adjacent layers into a sub-network. The partition is only applied to each sub-network, which reduces the dispersion of neurons from the adjacent layers. Finally, NeuMap employs a meta-heuristic algorithm to search for the best cluster-to-core mapping scheme in the narrowed search space. We evaluated NeuMap using six SNN-based applications. We showed that, compared to SpiNeMap and SNEAP, NeuMap reduces average energy consumption by 84% and 17% and has 55% and 12% lower spike latency, respectively. In this paper, the calculation of spike firing rate is only applied to the SNNs with a feed-forward topology such as spiking convolutional neural network.

In the future, we will exploit, such as the recurrent topology. In addition, other meta-heuristics algorithms, such as the hybrid harmony search algorithm [39], can be used to find the best mapping scheme. 

## Figures and Tables

**Table 1:** The SNN-based applications used to evaluate our approach.

SNNs	Topology	Neuron Model	Neurons	Synapses
MLP-Fashion-MNIST [30]	FeedForward(784,256,128,10)	LIF	1178	234,752
MLP-MNIST [34]	FeedForward(784,400,10)	LIF	1194	317,600
LeNet-5-MNIST [35]	CNN ^1^	IF	6598	286,120
CNN-Fashion-MNIST [30]	CNN ^2^	IF	7962	359,680
LeNet-5-CIFAR10 [35]	CNN ^3^	IF	11,166	659,024
CNN-CIFAR10 [7]	CNN ^4^	IF	12,266	971,776

^1^ Input(28 × 28) − [Conv, Pool] × 6 − [Conv, Pool] × 16 − FC(120) − FC(84) − FC(10); ^2^ Input(28 × 28) − [Conv, Pool] × 8 − [Conv, Pool] × 16 − FC(128) − FC(10); ^3^ Input(32 × 32 × 3) − [Conv, Pool] × 6 − [Conv, Pool] × 16 − FC(120) − FC(84) − FC(10); ^4^ Input(32 × 32 × 3) − Conv × 16 − [Conv, Pool] × 32 − Conv × 8 − FC(10).

**Table 2:** The comparison for two LSM networks.

	Total Spike Messages		Spike Latency (Normalized to SpiNeMap)		Energy Consumption on NoC (Normalized to SpiNeMap)	
	FSDD	NMNIST	FSDD	NMNIST	FSDD	NMNIST
Random partition	1,580,976	164,277	-	-	-	-
SpiNeMap [19]	1,580,976	164,277	100.00%	100.00%	100.00%	100.00%
SNEAP [20]	1,580,976	164,277	91.54%	99.24%	92.11%	95.62%
**NeuMap**	**1,580,976**	**164,277**	**72.09%**	**88.26%**	**55.54%**	**66.18%**

## Data Availability

The data presented in this study are available on request from corresponding authors.
